# High Dose, Prolonged Epsilon Aminocaproic Acid Infusion, and Recombinant Factor VII for Massive Postoperative Retroperitoneal Hemorrhage following Splenectomy

**DOI:** 10.1155/2016/1630385

**Published:** 2016-11-10

**Authors:** Alex T. Lee, Christopher R. Barnes, Shweta Jain, Ronald Pauldine

**Affiliations:** Department of Anesthesiology and Pain Medicine, University of Washington, Seattle, WA, USA

## Abstract

The antifibrinolytic agent *ε*-aminocaproic acid is used to decrease procedural blood loss in a variety of high risk surgeries. The utility of recombinant factor VII administration in massive hemorrhage has also been reported in a variety of settings, though the impact in a surgical context remains unclear. We describe the case of a patient who underwent massive open splenectomy and developed diffuse retroperitoneal bleeding on postoperative day one. Massive transfusion was initiated, but attempts to control hemorrhage with surgical and interventional radiology approaches were unsuccessful, as was recombinant factor VII administration. Commencement of a high dose aminocaproic acid infusion was followed by a prominent rise in fibrinogen levels and stabilization of the hemorrhage. Indications, dosages, and adverse effects of *ε*-aminocaproic acid as described in the literature are reviewed.

## 1. Introduction

*ε*-Aminocaproic acid is an antifibrinolytic agent used to decrease blood loss in surgeries with a high risk of severe hemorrhage. We present a case of massive bleeding occurring after open splenectomy. Multiple attempts to control the source surgically were unsuccessful, and bleeding continued despite massive transfusion of blood products and administration of recombinant factor VII. Initiation of a prolonged infusion of *ε*-aminocaproic acid led to resolution of the hemorrhage, resulting in delivery of a large cumulative dose without evidence of adverse effects.

## 2. Case Description

**Figure 1:**
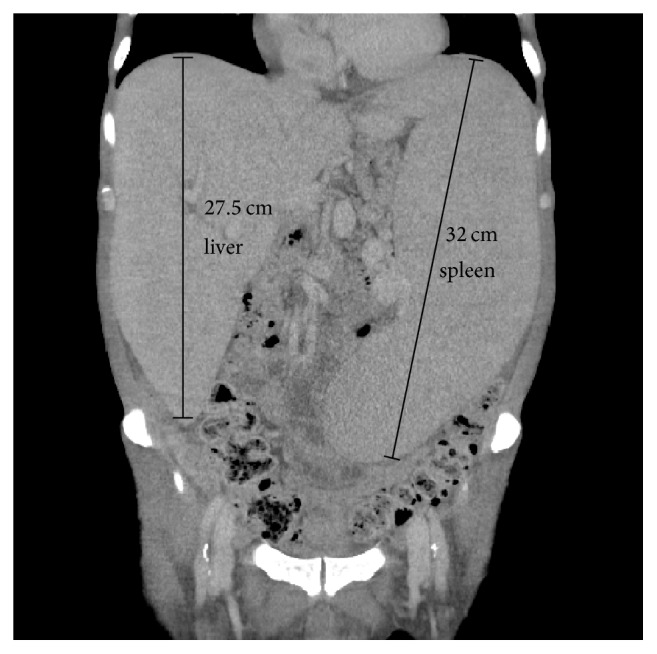
Preoperative CT demonstrating severe hepatosplenomegaly.

A 55-year-old man suffering from mixed myeloproliferative neoplasm (MPN) and myelodysplastic syndrome (MDS) who developed massive hepatosplenomegaly (Figure 1), anemia, and thrombocytopenia was scheduled to undergo palliative splenectomy. He had been treated with hydroxyurea for MPN/MDS overlap syndrome for years, before transforming to overt MDS with extramedullary hematopoiesis and excess blasts. His spleen had been enlarged to a craniocaudal length of 32 cm (Figure 1), causing abdominal pain, decreased oral intake, and a repetitive requirement for platelet transfusion. Removal of the organ was planned to relieve these symptoms while evaluation for allogeneic bone marrow transplantation was underway.

Due to anticipated heavy intraoperative hemorrhage, interventional radiology consultation was sought and a splenic artery embolization was performed a day preoperatively. Hematology consultation was also obtained by the surgical service, to address whether the patient's hematologic diagnosis might increase his risk of a consumptive coagulopathy to complicate the already high risk for surgical bleeding and if this could be addressed with any preoperative therapeutic intervention. Hematology consultants recommended commencement of a low dose *ε*-aminocaproic acid (E-ACA) infusion at a rate of 1 g every 6 hours to antagonize anticipated fibrinolysis. After transfusion of two units of platelets and two units of packed red blood cells (PRBCs) prior to surgery, his platelet count was 42,000/*μ*L with a hematocrit (Hct) of 25.9.

Intraoperatively the patient sustained an estimated blood loss of three liters and he received platelets, PRBCs, and plasma. Postoperatively he remained intubated due to concern for large fluid shifts resulting in airway and facial edema. He was transferred to the surgical intensive care unit (SICU). Three intraperitoneal Jackson-Pratt drains were placed during the procedure. His postoperative Hct was 24.4.

On the evening of postoperative day (POD) 1 the patient became hypotensive with systolic blood pressure in the 60–70 mmHg range. Several hundred milliliters per hour (mLs/hr) of frank bloody output came from the surgical drains and his hematocrit decreased to 19. Massive transfusion with PRBCs and FFP was initiated, and the patient was taken emergently to interventional radiology for attempt at further embolization of the mesenteric arterial circulation. However, no extravasation of blood was found from any arterial source and no embolization targets were identified. The patient returned to the SICU where he continued to have profuse bloody drainage averaging 200 mLs/hr. On the morning of POD 2 he received emergent exploratory laparotomy. Surgical exploration revealed diffuse retroperitoneal hemorrhage from extensive friable tissue and small vessel venous injury along the splenic bed, confirming lack of a major arterial source. Despite diffuse small vessel cautery and ligation throughout the retroperitoneal surface, he continued to produce 100–200 mL/hr of bloody fluid into the JP drains. At this point his transfusion regimen included 14 units of PRBCs, 9 units of fresh frozen plasma (FFP), 4 units of platelets, 1 unit of cryoprecipitate and 5 mg of recombinant factor VII.

Following a recommendation from consulting hematologists, a second dose of recombinant factor VII was given. When bleeding persisted, a 5 g E-ACA loading dose was administered two hours later followed by a 1 g/hr continuous infusion. Within the next few hours, there was a decrease in the bloody surgical drain output to less than 50 mLs/hr. Plasma fibrinogen levels rose from 211 mg/dL to 475 mg/dL over the next day without any additional transfusion of FFP or cryoprecipitate (Table 1). The high dose E-ACA infusion was continued through all of POD 3, during which only one additional PRBC and two platelet units were transfused. Fibrinogen levels continued to be supranormal ranging between 504 and 541 mg/dL. By POD 4 the hemorrhage resolved as hematocrit stabilized at 27-28 and drain output was minimal. No further blood products were transfused. At this point, E-ACA was decreased to 1 g q3 h. On POD 5 E-ACA was discontinued and the patient was successfully extubated. Fibrinogen levels decreased to a normal value of 327 mg/dL within 4 hours after termination of the E-ACA infusion. Renal function remained remarkably intact despite the hemodynamic instability and acute anemia that had occurred in the preceding days, as evidenced by a creatinine level of 0.37 mg/dL on POD 5. He was transferred out of the SICU on POD 7 and recovered uneventfully thereafter. No thromboembolic sequelae developed during hospitalization. He underwent allogeneic stem cell transplantation for MPN/MDS approximately a month after his discharge from intensive care.

## 3. Discussion

*ε*-Aminocaproic acid is a lysine analogue first described in the literature in 1957 for its antifibrinolytic properties [1]. It is one of the two lysine analogue fibrinolysis inhibitors used widely in current practice to decrease intraoperative blood loss for a variety of surgeries with elevated risk for significant hemorrhage, the other being tranexamic acid (TXA). A third antifibrinolytic agent, aprotinin, formerly enjoyed wide use particularly in cardiac surgery but was withdrawn by its manufacturer in 2008 after studies suggesting an association with increased incidence of myocardial infarction [2] and postoperative mortality [3].

E-ACA exerts its therapeutic action via inhibition of the serine protease plasmin, the principle agent in the native fibrinolytic process. Lysine analogues such as E-ACA and TXA are exogenous competitive inhibitors of plasmin's proteolytic degradation of fibrin and also competitively inhibit the activity of plasminogen activators [4]. These analogues appear to bind to sites on plasminogen that would ordinarily attach to lysine residues on fibrin. The overall result is lower rates of fibrin dissolution and a more stable pace of thrombogenesis.

The 2015 American Society of Anesthesiologists' (ASA) guidelines on perioperative blood loss management ascribes a Category A1 level of evidence (sufficient randomized controlled trials to support meta-analysis) favoring the use of intraoperative antifibrinolytic therapy in the perioperative setting to decrease blood loss and blood product transfusions in major cardiac, major orthopedic, and liver surgery [5]. A Cochrane meta-analysis suggests that the attributable decrease in intraprocedural blood loss for E-ACA in surgery with cardiopulmonary bypass is approximately 200 mLs and that the aggregate incidence of allogeneic blood transfusion in major procedures is reduced by 19% with its use [6]. In the orthopedic surgery literature, TXA has been studied more frequently than E-ACA; however, available evidence points favorably towards comparable efficacy and safety for E-ACA in knee, hip, and spine procedures [7–11]. The dosing of E-ACA varies considerably in the literature; commonly reported are loading doses ranging from 25 to 150 mg/kg followed by maintenance doses of 12.5 mg–30 mg/kg/hr [12].

Risks associated with lysine analogues include renal dysfunction and seizures. Lysine analogues are structurally similar to the neurotransmitter GABA, which is hypothesized to be an underlying mechanism for their potential to lower seizure threshold. Current data suggests that E-ACA is associated with fewer postoperative seizure complications than TXA [13, 14], while a meta-analysis of mostly adult patients suggested no difference in renal risk compared to TXA [6].

Concern exists that antifibrinolytic agents can precipitate thromboembolic disease, but the risk appears to be small and is unsubstantiated by any quality evidence. A 2011 Cochrane meta-analysis concluded that the literature does not support any increased incidence of myocardial infarction, stroke, deep vein thrombosis, or pulmonary embolism with the use of E-ACA compared to nonuse of any antifibrinolytic agent in cardiac surgery [6].

Our case illustrates a number of the salient characteristics of E-ACA in therapeutic application. Despite efforts to obtain hemostasis of a diffusely bleeding retroperitoneal bed through splenic artery embolization, surgical reexploration with small vessel ligation, transfusion of plasma products, and multiple doses of recombinant factor VII, our patient continued to have uncontrolled severe postoperative hemorrhage after open total resection of an enlarged spleen. Commencement of a prolonged, high dose E-ACA infusion (ultimately over a 48-hour period) was closely associated in time with stabilization of massive bleeding from diffuse microvascular sources, a hemorrhage that prior measures had failed to abate. While we cannot entirely exclude that factor VII, a dose of which had been given two hours prior to this, contributed also to achieving hemostasis, there are several reasons why we believe the effect can be largely attributed to E-ACA. One is that a first dose of factor VII given the evening before had not succeeded in slowing the rate of hemorrhage; likewise, bleeding persisted in the interval after factor VII was redosed and continued until after E-ACA was started.

It is worth noting also that the half-life of factor VII in plasma is approximately 8 hours, much less than the duration of our E-ACA infusion, and that the factor is essentially immediately bioavailable when given intravenously. Thus, the failure to achieve adequate hemostasis in the first hours after the two boluses of factor VII likely points to treatment failure or insufficiency overcome only when the E-ACA was administered. Furthermore, the decrement in bleeding was also associated with a rise in the patient's fibrinogen levels; this is a known effect of E-ACA that has been observed with human administration [15, 16], and its presence supports the contention that the onset of changes in the coagulation system with E-ACA administration helped mediate the hemorrhagic resolution that we observed clinically. While the exact mechanism for the elevation in fibrinogen level with E-ACA has never been clearly elucidated, we could hypothesize this to be a result of the lysine analogue's effect in antagonizing the breakdown of polymerized fibrin by plasmin, which in turn decreases the exposure of the circulation to areas of tissue injury that would trigger the contact activation pathway for thrombin activity. This decreased activation of thrombin could in turn lead to decreased conversion of fibrinogen into fibrin polymer and elevation of plasma fibrinogen levels. We acknowledge that one of the limitations of our conclusion about the role of E-ACA in this case is the lack of a more detailed coagulation assay, such as thromboelastography (TEG), that might have more clearly exposed some of the changes brought on by antifibrinolytic therapy. We do note, however, that TEG analysis is not routinely used in most clinical situations to guide antifibrinolytic administration. In this case, our decision to taper and ultimately discontinue the drug was guided clinically by a decrease of the bloody drainage output, lack of further need for blood product transfusion, and stability in the hematocrit level over several hours after the last PRBC transfusion.

Our case is notable for illustrating the crucial contribution that antagonizing the fibrinolytic pathway can have in treating severe hemorrhage from diffuse, surgically uncontrollable sources. Also interesting is that, in our patient, the cumulative dose of E-ACA over 5 days amounted to over 70 grams, substantially greater than the typical 10–25 g given in the cardiac surgery setting. While thromboembolic sequelae are a dreaded potential complication of antifibrinolytic therapy, our experience here is in accord with a general lack of findings in the wider clinical literature to suggest that E-ACA substantially increases pulmonary, coronary, cerebrovascular, or venous thromboembolism. In our case, a large dose of E-ACA was administered for a particularly precarious state of hemorrhage with poor prospects for interventional source control, with no resulting adverse hematologic, neurologic, or renal outcomes.

In conclusion, we would urge providers to consider targeting the fibrinolytic pathway through an antifibrinolytic agent such as E-ACA in situations of massive hemorrhage from diffuse bleeding sources that are poorly amenable to procedural ligation. Augmentation of the coagulation cascade with recombinant factor VII, such as that we used in this case, while not strictly evidence based, may be of utility also in these difficult to salvage scenarios. The possibility of inadvertent overtreatment and precipitation of thrombotic disease is a rational concern; however, the literature supports the safety of E-ACA in high risk surgical bleeding. Our case illustrates that even large cumulative doses of the drug can be administered safely with good efficacy. Furthermore, while the preponderance of clinical data on E-ACA consists of studies on intraoperative administration to decrease procedural blood loss, we have here presented a case where it was successfully used to treat postoperative hemorrhage. We surmise that further studies may be warranted to evaluate the potential role of antifibrinolytic therapy in applications beyond the operating room setting.

## Figures and Tables

**Table 1:** Postoperative transfusion requirements and degree of hemorrhage.

	Hematocrit (%)	Platelets (K/*μ*L)	INR	Fibrinogen (mg/dL)	Postoperative blood products (cumulative)		
					PRBC (units)	FFP (units)	Plt (units)
*Baseline* (2 hours preop)	25.9	42	1.2	361			
*Postoperative hour*							
12 hours	24.4	76	1	363	0	0	0
20 hours	23.3	62	1.1		2	0	1
28 hours^1^	19.1	39	1.3	259	6	2	2
36 hours	17	61	1.4	211	9	5	4
44 hours^2^	25.9	70	1.3	282	14	9	4
52 hours^3^	25.9	99	1.1	341	16	9	5
60 hours	24.7	73	1.3	388	18	10	5
68 hours	26.5	46	1.2	475	19	10	5
76 hours^4^	28	117	1.2	541	19	10	6
100 hours^5^	28.2	132	1.1	327	19	10	6

^1^Massive hemorrhage identified. Factor VII given.

^2^2nd dose of factor VII and 5 g aminocaproic acid load.

^3^Continuous aminocaproic acid infusion at 1 g/hr.

^4^Aminocaproic acid infusion decreased to 1 g/3 hrs.

^5^Four hours after aminocaproic acid infusion discontinued.

## References

[1] Alkjaersig N., Fletcher A. P., Sherry S. (1957). *ε*-aminocaproic acid: an inhibitor of plasminogen activation. *The Journal of Biological Chemistry*.

[2] Mangano D. T., Tudor I. C., Dietzel C. (2006). The risk associated with aprotinin in cardiac surgery. *The New England Journal of Medicine*.

[3] Fergusson D. A., Hébert P. C., Mazer C. D. (2008). A comparison of aprotinin and lysine analogues in high-risk cardiac surgery. *The New England Journal of Medicine*.

[4] Gans H., Krivit W. (1962). Problems in hemostasis during open-heart surgery: III. epsilon aminocaproic acid as an inhibitor of plasminogen activator activity. *Annals of Surgery*.

[5] American Society of Anesthesiologists Task Force on Perioperative Blood Management (2015). Practice guidelines for perioperative blood management: an update report by the American Society of Anesthesiologists Task Force on Perioperative Blood Management. *Anesthesiology*.

[6] Henry D. A., Carless P. A., Moxey A. J. (2011). Anti-fibrinolytic use for minimising perioperative allogeneic blood transfusion. *Cochrane Database of Systematic Reviews*.

[7] Kagoma Y. K., Crowther M. A., Douketis J., Bhandari M., Eikelboom J., Lim W. (2009). Use of antifibrinolytic therapy to reduce transfusion in patients undergoing orthopedic surgery: a systematic review of randomized trials. *Thrombosis Research*.

[8] Camarasa M. A., Ollé G., Serra-Prat M. (2006). Efficacy of aminocaproic, tranexamic acids in the control of bleeding during total knee replacement: a randomized clinical trial. *British Journal of Anaesthesia*.

[9] Harley B. J., Beaupré L. A., Jones C. A., Cinats J. G., Guenther C. R. (2002). The effect of epsilon aminocaproic acid on blood loss in patients who undergo primary total hip replacement: a pilot study. *Canadian Journal of Surgery*.

[10] Thompson G. H., Florentino-Pineda I., Poe-Kochert C., Armstrong D. G., Son-Hing J. P. (2008). The role of amicar in same-day anterior and posterior spinal fusion for idiopathic scoliosis. *Spine*.

[11] Gill J. B., Chin Y., Levin A., Feng D. (2008). The use of antifibrinolytic agents in spine surgery: a meta-analysis. *Journal of Bone and Joint Surgery A*.

[12] Ortmann E., Besser M. W., Klein A. A. (2013). Antifibrinolytic agents in current anaesthetic practice. *British Journal of Anaesthesia*.

[13] Keyl C., Uhl R., Beyersdorf F. (2011). High-dose tranexamic acid is related to increased risk of generalized seizures after aortic valve replacement. *European Journal of Cardio-thoracic Surgery*.

[14] Martin K., Knorr J., Breuer T. (2011). *Seizures* after open heart surgery: comparison of *ε*-aminocaproic acid and tranexamic acid. *Journal of Cardiothoracic and Vascular Anesthesia*.

[15] Naeye R. L. (1962). Thrombotic state after a hemorrhagic diathesis, a possible complication of therapy with epsilon aminocaproic acid. *Blood*.

[16] Thompson G. H., Florentino-Pineda I., Armstrong D. G., Poe-Kochert C. (2007). Fibrinogen levels following Amicar in surgery for idiopathic scoliosis. *Spine*.

